# Pre-Leukemic States: United by Difference

**DOI:** 10.3390/cancers13061382

**Published:** 2021-03-18

**Authors:** Christopher I. Slape

**Affiliations:** University of Queensland Diamantina Institute, University of Queensland, Brisbane 4072, Australia; c.slape@uq.edu.au

Pre-leukemia is a catch-all term for any haematological condition which predisposes the individual towards developing leukemia. The development of any cancer begins long before any clinically detectable sign is apparent. It requires a series of driver acquisition events, each providing a growth or survival advantage to a cell such that it will expand. Each clonally selected event is followed by another, until eventually, the clone reaches the definition of what we call cancer. It is sometimes difficult to discriminate cancer from non-cancer, and experts will disagree in some cases on whether a particular sample is cancerous or pre-malignant. What is clear, however, is that all cancers pass through some pre-malignant state. In most cancers, this process is not clinically observed and therefore, poorly studied (there are of course exceptions—Barrett’s oesophagus [1] and the transition from naevi to melanoma [2] are two such exceptions). In the haematological system, some of these pre-malignant conditions prominently present clinically, affording us greater opportunity to study and learn from them.

Among the pre-leukemic states which present clinically, there are two major categories: the myelodysplastic syndromes (MDS) and the myeloproliferative neoplasms (MPN). MDS are characterised by ineffective haematopoiesis, leading to cytopenia in multiple blood cell lineages, and are also associated with dysplasia suggesting differentiation abnormalities. They are a very heterogeneous group of diseases with multiple recurrent cytogenetic abnormalities and are generally considered to be the result of aberrant hematopoietic stem cell function, with deficiencies in the ability to differentiate [3]. MPN are characterised by the expansion of the terminally differentiated myeloid cells and are also the result of aberrant hematopoietic stem cell function [4]. They are subdivided into three main categories: polycythaemia vera, essential thrombocythemia and primary myelofibrosis. Polycythaemia vera is almost always driven by the presence of a *JAK2* V617F mutation [5], and essential thrombocythemia and primary myelofibrosis usually also feature disrupted JAK-STAT signalling [6]. There are other MPNs that fall outside these three categories, including chronic myeloid leukemia, which is classified as an MPN by the WHO [7]. Both MDS and MPN are fairly indolent diseases, which creates a substantial health care burden.

Some pre-leukemic states do not present clinically but have either been observed from clinical data or experimental models, or their existence in patients has been inferred retrospectively. A prominent example of the former is Clonal Haematopoiesis of Indeterminate Potential (CHIP), a state of reduced clonal diversity in the haematopoietic output with no morphological abnormality [8,9]. The extent to which this predisposes an individual towards developing leukemia is debatable, but there is at least some effect [10]. Experimentally, transgenic mouse models of T cell leukemia with a long latency have been demonstrated to have a phenotypically defined pre-leukemic state, where the thymocytes can be transplanted and expand in a recipient animal [11]. An example of the retrospective inference of pre-leukemia is from twin studies which identified identical mutations in twins who developed B cell acute lymphoblastic leukemia later in life, necessitating that the mutation had occurred in utero and therefore, the patients had carried this pre-leukemic clone their entire life [12]. All of these examples support the general consensus that, even if no clinical condition is observed, every leukemia is preceded by a pre-leukemic state.

A focus on pre-leukemia is important for a number of reasons. The clinical burden of the clinically presenting pre-leukemias is large, as the diseases can be quite indolent and require supportive care; meanwhile, curative therapies are lacking. The risk of transformation carries with it a very poor prognosis for patients. Additionally, the access to samples and data from the haematological pre-malignancies may be informative for the less studied pre-malignancies of solid tissues, affording haematological research to once again lead the way. The study of pre-leukemia will therefore lead to new insights into the origin of leukemia, and cancer more broadly. This Special Issue highlights new research in the area of pre-leukemia and presents reviews on different aspects of the topic.
